# The influence of lifestyle interventions and overweight on infertility: a systematic review, meta-analysis, and meta-regression of randomized controlled trials

**DOI:** 10.3389/fmed.2023.1264947

**Published:** 2023-11-01

**Authors:** Ana Sustarsic, Vedran Hadzic, Cécil J. W. Meulenberg, Ensar Abazovic, Mateja Videmsek, Tanja Burnik Papler, Armin H. Paravlic

**Affiliations:** ^1^Faculty of Sports, University of Ljubljana, Ljubljana, Slovenia; ^2^Science and Research Center Koper, Institute for Kinesiology Research, Koper, Slovenia; ^3^Faculty of Sport and Physical Education, University of Sarajevo, Sarajevo, Bosnia and Herzegovina; ^4^Division of Gynecology, Department of Human Reproduction, University Medical Center Ljubljana, Ljubljana, Slovenia; ^5^Faculty of Sports Studies, Masaryk University, Brno, Czechia

**Keywords:** physical activity, infertility, intervention, overweight, pregnancy, ovulation

## Abstract

This study aimed to investigate the effect of lifestyle intervention (LSI) on diagnosed infertility in overweight and obese women. A systematic review and meta-analysis were conducted. A literature search was performed on the following databases from September 2022 to December 2022: PubMed, Web of Science, and SPORTDiscus. The inclusion criteria were the following: women between 18 and 45 years of age, BMI over 25.0 kg/m^2^, diagnosed with infertility, a weight loss intervention, and control group part of RCTs. In total, 15 studies were identified and included. The meta-analysis shows a beneficial effect of LSI on reducing weight, waist circumference, and BMI and increasing infertility. A significantly beneficial effect of lifestyle intervention on weight reduction was observed for participants who initially had a higher BMI, while a non-significant effect was observed for individuals with a BMI above 35 kg/m^2^. The meta-analysis showed a beneficial effect of lifestyle intervention on ovulation incidence and sex hormone-binding globulin. The lifestyle intervention group had 11.23 times more ovulatory incidence than the control group, which in turn increased the ability to conceive. As robust evidence for the effect of lifestyle interventions on infertility in obese and overweight women was found, it is advised to integrate similar interventions into future infertility treatment processes.

## Introduction

Infertility is a medical state generally defined as a failure to conceive after 12 months of regular intercourse. Infertility is a rising problem in human society, and although the prevalence worldwide has been difficult to ascertain with limited population-based studies and inconsistent clinical definitions, it is estimated to affect between 8 and 12% of reproductive-age couples (1). There are no exact data for Slovenia, but it is estimated that the situation is comparable to that in other European countries, which means that every 8 out of 12 couples face fertility problems (2). Primary infertility means that a couple has never achieved pregnancy, whereas secondary infertility means that a couple has had at least one prior successful conception (3).

Causes of infertility can be found in both female and male partners of reproductive-age couples. In 40–50% of the cases, the cause of infertility can be found in the reproductive system of the female partner, while in 30–40%, the cause of infertility is found in that of the male partner, and in 10% of cases, the cause is found in that of both partners. However, in 10% of couples, the cause of infertility remains unknown—idiopathic infertility (4). In women, as much as 80% of infertility can be attributed to three causes: endometriosis (5), tubal factor infertility, and polycystic ovary syndrome (PCOS) (6). Besides, a couple’s lifestyle (inactivity, stress, unsuitable diet), smoking habits (6–8), excessive consumption of alcohol (6, 7) and coffee (7), environmental pollutants (9), and psychological factors (6) can play major roles in human fertility. Excessive body weight is also an important cause of infertility (6, 10–12) and may trigger certain factors that negatively affect infertility (abnormal metabolism, hormonal disorders, menstrual and ovulary disorders, PCOS, hyperinsulinemia, hyperandrogenism, etc.) (11, 12).

The prevalence of obesity and overweight is rising worldwide and has a detrimental effect on different functions of the human body, including reproduction. In particular, obese women suffer from hormone disorders, which lead to menstrual dysfunction, anovulation, and, consequently, infertility. In women with PCOS, hormone disorders and subfertility are common, while with additional obesity, the adipocytes begin to function as endocrine organs (13). A higher BMI is associated with a poorer fertility prognosis and simultaneously shows poorer reproductive results, regardless of the method of conception. Furthermore, a high BMI leads to a higher miscarriage rate, poor pregnancy outcomes, a higher risk of complications during pregnancy, and impaired fetal wellbeing (14). It was found that weight reduction in obese and overweight women improves reproductive outcomes by ameliorating fertility, regularizing menstrual cycles, and increasing the chance of spontaneous ovulation and conception in anovulation (11, 12, 15).

Various approaches are used to reduce weight, including interventions that change lifestyle habits such as applying regular sports activities as well as nutritional and psychological counseling, while drugs that can contribute to weight loss have been used less frequently. Various studies have shown that weight-loss lifestyle-changing interventions in overweight and obese women have a positive effect on hormonal and metabolic factors. These interventions affect the levels of fasting glucose, insulin, androstenedione, testosterone, anti-Mullerian hormone, estrogen, the homeostasis model assessment of insulin resistance (HOMA-IR), and sex hormone-binding globulin (SHBG) (16–18). Lifestyle interventions (LSI) also increase the rate of spontaneous as well as *in vitro* fertilization (IVF) pregnancies and the number of live births (19–21).

Physical activity (PA) has an important role during preconception, pregnancy, and postpartum. Well-balanced PA and energy state have fundamentally been related to an optimal reproductive system and good general health (22). It is necessary to consider the intensity and frequency of exercise because excessive exercise can have a negative effect on fertility. However, several studies have confirmed the positive effect of regular and moderate PA on fertility in women. A systematic review by Hakimi and Cameron has shown that exercise, with or without diet, can lead to a resumption of ovulation in overweight/obese women suffering from PCOS or anovulatory infertility (23). A prospective cohort study investigated the relationship between PA and time to pregnancy. In this study, moderate PA was associated with a small increase in fecundability, regardless of BMI. These findings indicate that PA of any type might improve fertility among overweight and obese women, a subgroup at higher risk of infertility (24).

In a recent meta-analysis, the effect of PA on the reproductive health of young women was analyzed (15). However, in that particular study, there were no data on which intervention and PA might have the best results or the greatest effect of the included components. Moreover, no data on PA frequency, intensity, or duration were reported. PA is an important factor in weight loss, which is sometimes underestimated, but it is necessary to realize that not all forms of PA are suitable for obese people. Thus, to establish more detailed associations between weight-loss lifestyle-changing interventions and infertility, the present study performed a meta-analysis with the inclusion of recent studies that clearly stated the abovementioned relevant LSI parameters. Potential results from effective detailed PA interventions would be very relevant to integrate as evidence-based recommended LSI within the health system, specifically in the treatment of infertility in overweight and obese women.

## Materials and methods

### Search strategy and study selection

The literature search was conducted from September 2022 to December 2022. The following databases were examined: PubMed, Web of Science, and SPORTDiscus. The word AND was used between the main groups of keywords related to infertility (“infertility”, “sterility”, “subfertility”, “*in vitro* fertilization”, “IVF”), gender (“female”, “women”), weight (“obesity”, “overweight”), and intervention (“weight reduction”, “lifestyle”, “healthy lifestyle”, “lifestyle intervention”, “intervention”, “physical activity” or “training”), and the word OR was used between the keywords within the group.

**Figure 1:**
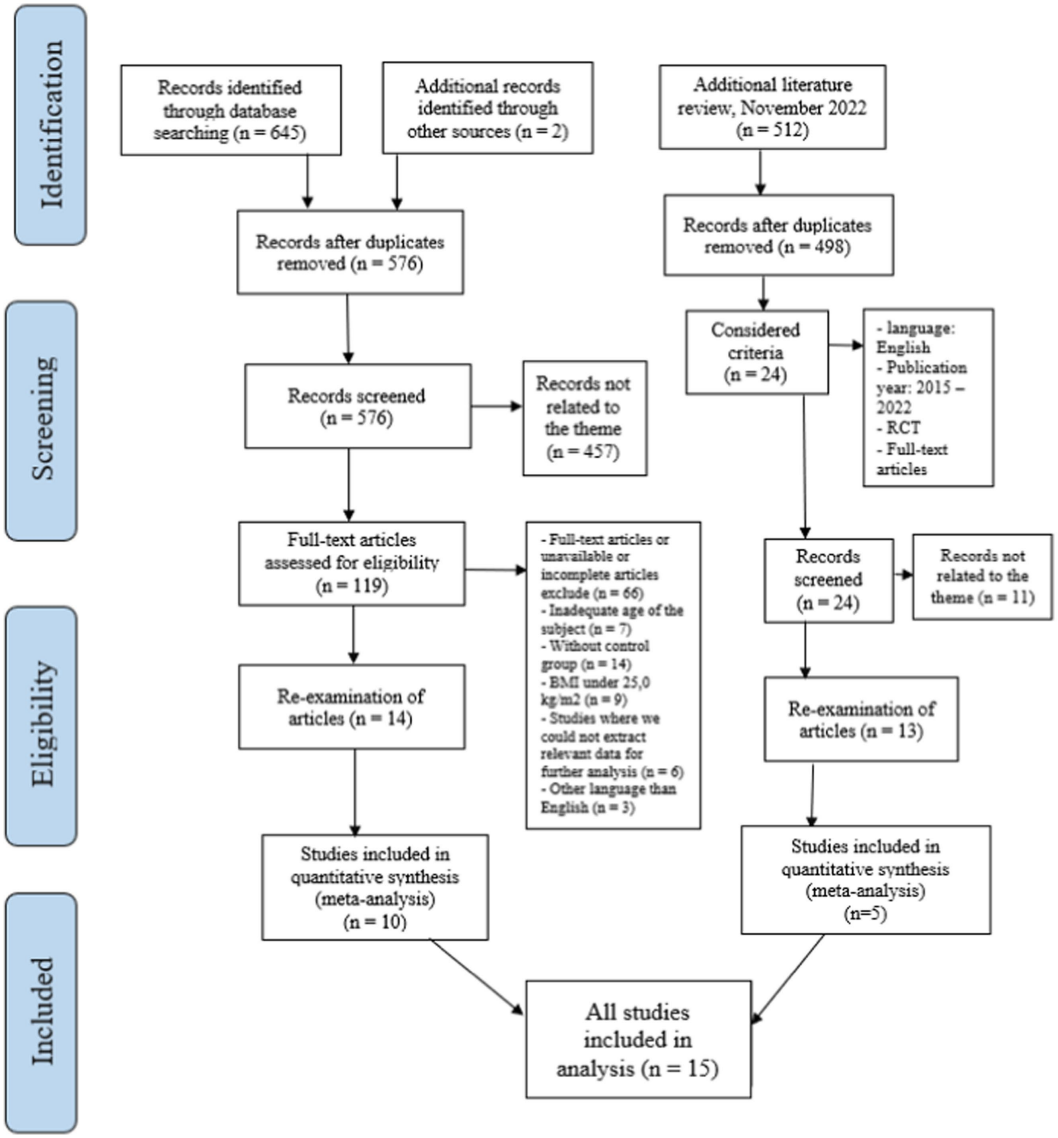
PRISMA flow diagram of the study of the first and second selection processes.

The first review of study titles and abstracts was conducted by the first reviewer (AS), and the final review and selection were conducted by another reviewer (AP). Eligible studies that passed the selection process were included according to the determined inclusion and exclusion criteria. The inclusion criteria were the following: women between 18 and 45 years of age, BMI over 25.0 kg/m^2^, diagnosed with infertility or PCOS or both, a weight loss intervention, a control group, availability in the full-text English language, and research design RCTs. In the final review of the selected articles, studies that did not have results for further and definitive analysis were excluded. The study selection process is illustrated in Figure 1 following Preferred Reporting Items for Systematic Reviews and Meta-analyses (PRISMA) guidelines (25).

The primary outcome measures included ovulation improvement, pregnancy rates, and live birth rates, while the secondary outcome measures included changes in weight, BMI, waist circumference, and hormonal and blood factors.

### Data extraction

The methodological quality of the included studies was assessed using the PEDro scale (26) by two reviewers independently (EA and AP). The PEDro scale comprises 11 items designed to rate methodological quality (26). Each satisfied item contributes 1 point to the overall PEDro score (range 0–10 points). However, item 1 (indicate briefly pertaining to external validity) was not included as part of the study quality rating for this review because it pertains to external validity, which was beyond the scope of the current review questions. Additionally, the Template for Intervention Description and Replication (TIDieR) checklist was used to assess the completeness of the intervention descriptions for both the experimental and control groups (27). The quality of evidence was assessed using the Grading of Recommendations Assessment, Development and Evaluation (GRADE) system, where classifications were made as follows: “high quality,” “moderate quality,” “low quality,” and “very low quality” (28). However, several reasons might lead to the degradation of the quality of the evidence (28). Thus, in the current study, the following criteria were considered when assessing confidence in evidence: design limitation (if the majority of studies in the meta-analysis had a PEDro score of <6); imprecision based on small sample size [< 300 for each pooled outcome (29)]; and inconsistency of the results (substantial heterogeneity within effect estimates, I2 ≥ 50%). This review did not consider the indirectness criterion because the eligibility criteria ensured a specific population with relevant outcomes.

### Statistical analysis

The meta-analyses were performed using Comprehensive Meta-analysis software (version 2.0; Biostat Inc., Englewood, NJ, United States). Except for ovulation and pregnancy, for all reported outcome measures, the difference in means (DM) and 95% CIs were calculated and presented in their respective units. Thus, weight was presented in kilograms (kg), BMI in kg/m^2^, waist circumference in centimeters (cm), blood glucose in mmol/l, blood insulin in milli mass units per liter (mU/L), SHBG and testosterone (nmol/L), and FAI index (no unit). HOMA-IR was calculated by multiplying fasting serum insulin (μU/ml) and fasting plasma glucose (mmol/L) in arbitrary units. The odds ratio (OR) was reported for ovulation, pregnancy, and live births. The random-effects model of the meta-analysis was applied in all comparisons to determine the effect of the intervention on measures of interest. To investigate the effects of BMI on weight management and waist circumference, a subgroup analysis was performed by comparing groups with initially lower (i.e., <35 kg/m^2^) and greater (i.e., ≥35 kg/m^2^) BMI, respectively.

Furthermore, a random-effects meta-regression was performed to examine whether the effects of LSI on weight and pregnancy were moderated by the initial age and BMI of the participants, as well as different training variables. Training variables were grouped according to the following: training volume (i.e., period, weekly frequency, and the total number of training sessions) and time spent in training (i.e., duration of a single training session). To minimize the risk of overfitting, a meta-regression was performed when a minimum of 10 studies were eligible per examined covariate (30).

The publication bias was assessed by examining the asymmetry of the funnel plots using Egger’s test, and a significant publication bias was considered if the value of p was <0.10. The I^2^ statistic was used to investigate between-study heterogeneity, where values of 25, 50, and 75% represent low, moderate, and high statistical heterogeneity, respectively (31). Statistical significance was set at the level of a value of p of <0.05.

## Results

Egger’s test was performed to provide statistical evidence of funnel plot asymmetry. The results indicated no publication bias for the following meta-analysis: weight management (*p* = 0.497), waist circumference (*p* = 0.777), glucose (*p* = 0.732), insulin (*p* = 0.804), HOMA-R (*p* = 0.901), SHBG (*p* = 0.106), and FAI (*p* = 0.246), respectively. For all other analyses, the results indicated publication bias (*p* < 0.10).

### Study selection and characteristics

Following a systematic literature search in different databases, 15 studies were identified and included (Table 1). The trials included a mix of three study design types: seven RCTs, three randomized comparison trials, and five RCT pilot studies. The research covered different LSIs: diet, PA, pharmacological treatment, and psychological help.

**Table 1:** Characteristics of the included studies.

Study	Study design	Population (initial weight, BMI, sample size, A, PCOS, ART)	Duration	Intervention (PA, diet, PBA, PT)	PA intervention	Outcome measure (W, BMI, O, P, M, LB)	Results
Gallety et al. (45)	RCT	EX: 102.4 ± 18.9; NR; 32 CON: 101.9 ± 18.1; NR; 32 A: 26–36	24 weeks	EX: PA, diet, PBA CON: PA, diet, PBA, PT	60 min of group exercise, 1 t/w	W	EX: 97.5 ± 5.2 CON: 96.7 ± 5.2
Thomson et al. (39)	Randomized comparison trial	EX: 97.6 ± 18.4; NR; 31 CON: 102.1 ± 18.3; NR; 33 *3rd: 100.5 ± 18.1; 30* A: 18–41 PCOS	20 weeks	EX: PA, diet CON: PA, diet, *3rd: diet*	EX: aerobic training of 25–45 min of walking or jogging 5 t/w, intensity 60–80% HR max CON: aerobic training as EX, resistance training: 3×12, 2 t/w, 5 consisted resistance exercises: bench press, lat pulldown, leg press, knee extension, and sit-ups, progressive intensity (training load of 50–60% 1RM in the first 2 weeks and increased to 60–75% 1RM for the following weeks)	W, O, P, M	EX: W 87.5 ± 18.4, O 50.0% (3/6), P 3.2% (1/31), M 42.9% (9/21) CON: W 93.5 ± 18.4, O 42.9% (3/7), P 3.0% (1/33), M 44.4% (8/18) *3rd: 91.9 ± 18.6, O 50.0% (6/12), P 3.3% (1/30), M 21.4% (3/14)*
Palomba et al. (20)	Randomized comparison trial	EX: 85.3 ± 6.4; 31.3 ± 2.6; 32 CON: 86.2 ± 6.9; 32.3 ± 3.7; 32 *3rd: 87.0 ± 6.9; 31.1* ± 2.9; *32* A: 18–35 PCOS	6 weeks	EX: PA, diet CON: PA, diet, PT *3rd: PT*	Structured exercise training (30 min on a bicycle ergometer, 3 t/w), the intensity increased gradually until a target of 60–70% VO2max consumption was achieved (according to an initial cardiopulmonary exercise test)	W, BMI, O, P	EX: W 86.2 ± 6.9, BMI 28.9 ± 2.3, O 12.5% (4/32), P 0% (0/32) CON: W 81.8 ± 6.0, BMI 32.3 ± 3.5, O 37.5% (12/32), P 3.1% (1/32) *3rd: 86.3 ± 6.4, BMI 28.4* ± 2.5, *O 9.4% (3/32), P 0% (0/32)*
Moran et al. (51)	RCT, pilot study	EX: 93.0 ± 16.0; 34.0 ± 4.5; 18 CON: 92.1 ± 13.8; 33.9 ± 4.4; 20 A: 18–41 ART	5–9 weeks	EX: PA, diet CON: they got advice but no active follow-up	Progressive walking program (20–45 min, 3 t/w), resistance training (1–2, 8–10 reps, 2 t/w), moderate intensity	W, BMI, P, LB	EX: W 89.2 ± 3.0, BMI 32.6 ± 1.1, P 66.7% (12/18), LB 38.9% (7/18) CON: W 91.6 ± 1.2, BMI 33.7 ± 0.4, P 40.0% (8/20), LB 25.0% (5/20)
Sim et al. (48)	RCT	EX: 95.8 ± 12,7; 35.1 ± 3.8; 27 CON: 104.0 ± 16.1; 38.0 ± 5.2; 22 A: 18–37 ART	12 weeks	EX: PA, diet, PBA CON: they got advice but no active follow-up	Unsupervised increasing walking to a target of 10,000 steps over 6 weeks (measured by a pedometer) and then 6 weeks 10,000 steps; intensity light to moderate	W, BMI, LB	EX: W 89.2 ± 4.6, BMI 32.7 ± 1.6, LB 44.4% (12/27) CON: W 102.4 ± 3.6, 37.4 ± 1.3, LB 13,6% (3/22)
Legro et al. (18)	Randomized comparison trial	EX: 96.0 ± 15.8; 35.1 ± 4.6; 50 CON: 95.2 ± 14.5; 35.1 ± 4.6; 50 *3rd: 94.6 ± 14.4; 35.5* ± 4.4; *49* A: 18–40 PCOS	16 weeks	EX: PA, diet CON: PA, diet, PT *3rd: PT – control group*	Aerobic exercise (10 min/day of bris walking or similar aerobic activity for the first 5 days and gradually increased over 4 months to 30–35 min/day), 5 t/w, the goal is 150 min/week activity	W, BMI, P, LB	EX: W 89.9 ± 6.1, BMI 32.9 ± NR, P 26.0% (13/50), LB 26.0% (13/50) CON: W 89.1 ± 6.1, BMI 34.8 ± NR, P 26.0% (13/50), LB 24.0% (12/50) *3rd: W 93.5 ± 1.1, P 14.3% (7/49), LB 14.3% (7/49)*
Dokras et al. (47)	RCT	EX: 97.0 ± 15.5; 35.4 ± 4.6; 44 CON: 94.6 ± 15.0; 35.3 ± 4.2; 43 *3rd: 95.1 ± 1.4; 35.3* ± 4.4; *45* A: 27–42 PCOS	16 weeks	EX: PA, diet, PT (for losing weight) CON: PA, diet, PT (to improve infertility) *3rd: PT*	Aerobic exercise (10 min/day of bris walking or similar aerobic activity, began at 10 min for the first 5 days and gradually increased over 16 weeks to 30–35 min), 5 t/w, the goal is 150 min/week activity	W, BMI	EX: W 90.6 ± 6.4, BMI 33.3 ± 2.4 CON: W 88.2 ± 6.4, BMI 33.2 ± 2.4 3rd: 93.9 ± 1.2
Einarsson et al. (34)	RCT	EX: 92.4 ± 8.0; 33.1 ± 1.3; 152 CON: 91.0 ± 8.4; 32.9 ± 1.4; 153 A: 18–38 PCOS, ART	16 weeks	EX: PA, diet, PT CON: PT	All patients had scheduled individual visits with a health professional at weeks 0 (baseline), 2, 5, 8, and 12, where weight was recorded. Not reported type, frequency, and intensity of PA.	W, BMI, P, LB	EX: W 83.3 ± 6.8, BMI 29.8 ± 2.4, P 10.5% (16/152), LB 29.6% (45/152) CON: W 92.9 ± 1.9, BMI 32.5 ± 0.7, P 2.6% (4/153), LB 27.5% (45/153)
Becker et al. (40)	RCT	EX: 77.0 ± 2.0; 28.7 ± 0.6; 14 CON: 74.4 ± 2.7; 28.8 ± 1.0¸11 A: 18–35 ART	12 weeks	EX: diet CON: no intervention, advice	Maintain the same level of PA as before the intervention	W, BMI, P, LB	EX: W 72.5 ± 0.8, BMI 26.7 ± 0.5, P 21.4%, LB 21.4% CON: W 73.7 ± 0.7, BMI 29.1 ± 0.3, P 0.0%, LB 0.0%
Mutsaerts et al. (19)	RCT	EX: NR; NR; 290 CON: NR; NR; 287 A: 18–39 ART	24 weeks	EX: PA, die, PBA CON: no intervention. The patient went directly to IVF	Aerobic exercise (daily PA was stimulated with the use of a pedometer, aimed at 10,000 steps per day), at least 2 or 3 t/w, moderate intensity	P, LB	EX: P 53.6%, LB 27.1% CON: P 58.8%, LB 35.2%
Nagelberg et al. (44)	RCT, pilot study	EX: NR; NR; 10 CON: NR; NR; 11 A: 18–42 PCOS	4 weeks	EX: PA, diet CON: PA	Aerobic exercise (daily PA was stimulated with the use of a pedometer, aimed at 10,000 steps per day); exercise diary	O, P	EX: O 40.0%, P 40.0% CON: O 9.1%, P 27.3%
Espinos et al. (49)	RCT, pilot study	EX: 91.7 ± 11.8; NR; 21 CON: 89.2 ± 1.5; NR, 20 A: 29–37 ART	12 weeks	EX: PA, diet CON: no intervention. The patient went directly to IVF	Aerobic exercise (walking on a treadmill or pedaling stationary bicycles), 3 t/w, 60 min	W, O, P, LB	EX: W 85.3 ± 11.1, O 54.5%, P 57.1%, LB 61.9% CON: W NR, O NR, P 35.0%, LB 30.0%
Rothberg et al. (50)	RCT, pilot study	EX: 108.0 ± 10.0; 41.0 ± 4.0; 6 CON: 107.0 ± 14.0; 41.0 ± 4.0; 5 A: 18–40	12 weeks	EX: PA, diet CON: diet	Aerobic exercise (40 min/day, moderate PA)	W, BMI, O, P, LB	EX: W 94.0 ± 6.0, BMI 36.0 ± 2.0, O 50.0%, P 50.0%, LB 50.0% CON: W 102.0 ± 5.0, BMI 39.0 ± 2.0, O 0.0%, P 0.0%, LB 0.0%
Kiel et al. (41)	RCT, pilot study	EX: 85.7 ± 3.5; 28.9 ± 2.4; 8 CON: 87.9 ± 2.9; 31.2 ± 1.3; 10 A: > 18 ART	10 weeks	EX: PA, diet CON: gift card for the local gym for 85$	Resistance training (3 t/w, 2 times 4×4 min high-intensity training, third 10×1 min high-intensity training, 85–95% HRmax, walking or running on a treadmill)	W, P	EX: W 85.1 ± 4.7, BMI 29.6 ± 1.2, P 50–0% CON: W 87.2 ± 4.7, BMI 30.3 ± 1.2, P 44.0%
Legro et al. (42)	RCT	EX: 108.4 ± 22.7; 39.2 ± 7.0; 187 CON: 107.4 ± 20.8; 39.4 ± 6.9; 191 A: 18–40	16 weeks	EX: PA, diet CON: PA	Aerobic exercise (daily PA was stimulated with the use of a Fitbit activity tracker and a pedometer, aimed at 10,000 steps per day)	W, BMI, P, LB	EX: W 101.1 ± 6.0, BMI 36.6 ± 2.1, P 53.2%, LB 32.4% CON: W 107.3 ± 3.4, BMI 39.3 ± 1.3, P 48.2%, LB 37.2%

RCT, randomized controlled trial; EX, experimental group; CON, control group; BMI, body mass index; A, age; PCOS, women with polycystic ovary syndrome; ART, Assisted reproductive technology; NR, not reported; PA, physical activity; PBA, psychological/behavior advice; PT, pharmacological treatment; t/w, times per week; W, weight; O, ovulation; P, pregnancy; M, menstrual cycle; LB, live birth; 3rd, the third group.

^a^Assisted reproductive tehchnology (ART) includes all fertility treatments in which either eggs or embryos are handled (in vitro fertilization - IVF), intra- cytoplasmic sperm injection – ICSI, cycles or subsequent cryostored embryo transfer cycles). The main type of ART is IVF.

### Quality and completeness of reporting

The reported completeness of intervention reporting was higher for the experimental conditions (mean: 73%; range from 27 to 100%) than for the control groups (mean: 57%; range from 18 to 91%). Compared to previously published data about the completeness of intervention reporting in interventional studies (32), the current meta-analysis included studies with sufficiently detailed exercise program descriptions. Table 2 shows the summarized results of the GRADE system and the PEDro scale, both used for assessing the quality of evidence.

**Table 2:** Grades of recommendation, assessment, development, and evaluation (GRADE) for results summarized.

Outcome	Trials (n)	Participants (n)	Diff in Means	LLCI	HLCI	*I*^2^ (%)	PEDro score	Quality of evidence (GRADE)
Weight	12	1,205	−3.52	−6.57	−0.47	76	7	Moderate quality
BMI	9	977	−1.75	−2.60	−0.90	65	7	Moderate quality
WC	9	749	−3.34	−5.06	−1.63	26	7	High quality
Glucose	5	183	−0.19	−0.29	−0.09	0	7	Moderate quality
Insulin	5	551	−0.98	−2.23	0.28	2	7	Moderate quality
HOMA-IR	5	183	−0.01	−0.45	0.43	59	7	Moderate quality
SHBG	5	551	5.55	1.89	9.21	47	7	Moderate quality
FAI index	3	172	−0.53	−1.91	0.86	0	7	Moderate quality
Testosterone	5	551	0.12	0.02	0.23	0.	7	Moderate quality
Ovulation	4	123	11.23^*^	2.51	50.23	35	7	Moderate quality
Pregnancy	11	1,567	1.49^*^	1.04	2.15	44.	7	High quality
Live births	9	1,526	1.51^*^	0.92	2.47	65	7	Moderate

^*^ - data are presented as odds ratio; LCI – lower limit confidence interval; HLCI – higher limit confidence interval, I^2^ – test of heterogeneity.

**Figure 2:**
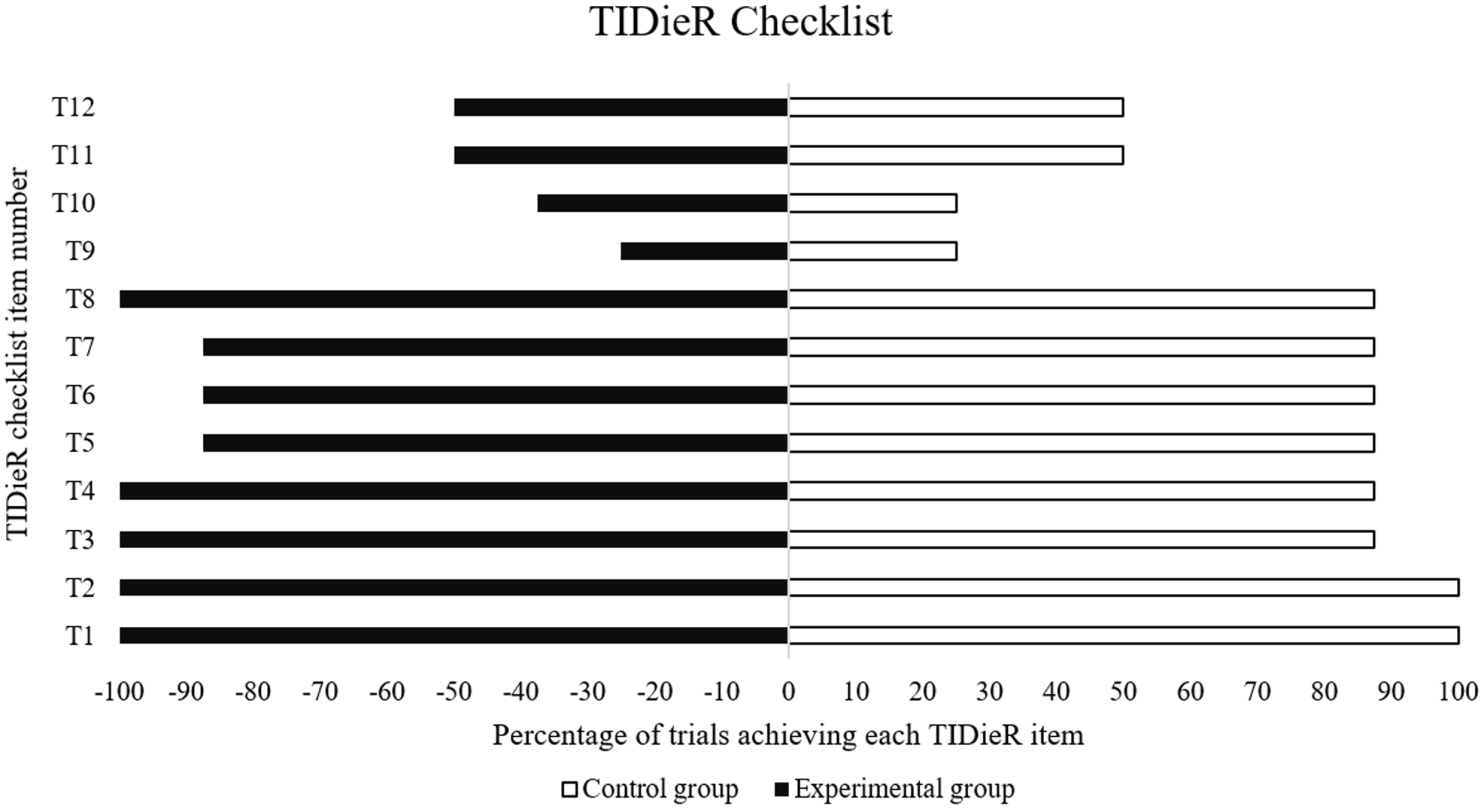
TIDieR checklist.

The TIDieR checklist (Figure 2) provides a systematic way to describe the intervention, including rationale, materials used, procedures, how, where, when, and by whom the training was provided, and how the training was tailored and modified.

### Effect of LSI on the anthropometric measures

#### Weight

**Figure 3:**
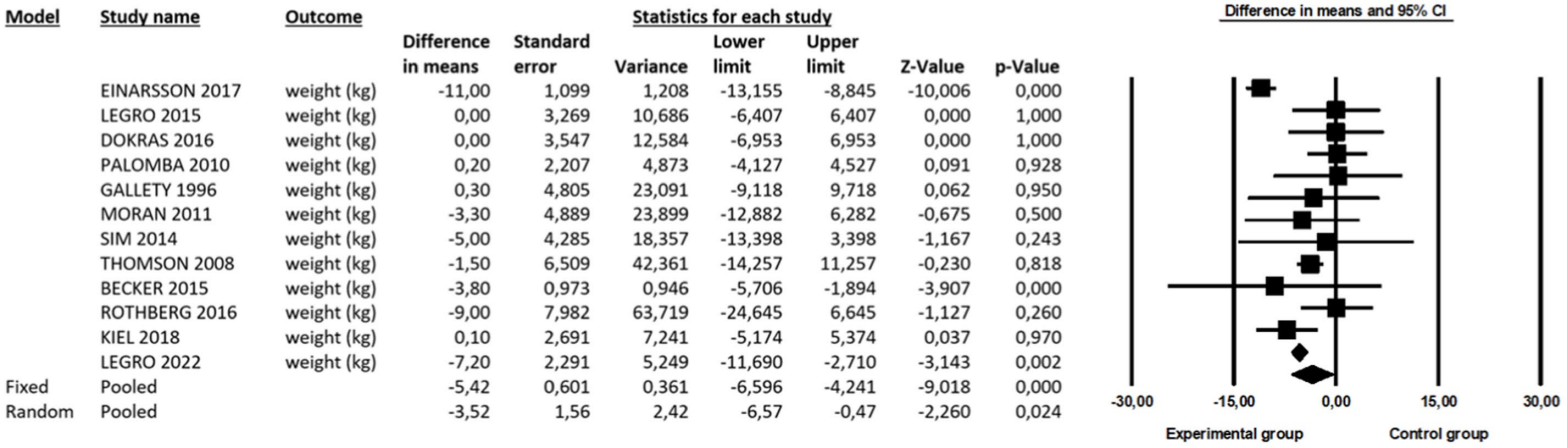
A beneficial effect of LSI on weight.

The current meta-analysis of twelve studies with a total of 1,205 patients showed a beneficial effect (DM = −3.52 kg, 95% CI -6.57 to −0.47, df = 11; *p* = 0.024) of weight management on infertility (Figure 3). The evidence was downgraded from high to moderate due to high heterogeneity (*I^2^* = 78%; *p* < 0.001). Owing to this substantial heterogeneity, sub-analysis and meta-regression analyses were performed. Sub-group analysis revealed that the effects of the interventions were not moderated by BMI (Q = 0.001; *p* = 0.980). In brief, a significantly beneficial effect was observed only for participants who had initially high BMI (DM = −3.69 kg, 95% CI -6.76 to −0.61, *n* = 6, *p* = 0.019) and not for those with less than 35 kg/m^2^ of BMI (DM = −3.62 kg, 95% CI -8.05 to 0.81, *n* = 6, *p* = 0.109).

#### BMI

The meta-analysis of nine studies with a total of 977 patients showed a beneficial effect (DM = −1.75 kg/m^2^, 95% CI -2.60 to −0.90, df = 8; *p* < 0.001) on BMI management. The evidence was downgraded from high to moderate due to moderate to high heterogeneity (I^2^ = 65%; *p* = 0.004). Hence, sub-analysis and meta-regression analyses were performed. Sub-group analysis revealed that the effects of the interventions were not moderated by BMI (Q = 0.081; *p* = 0.776). In brief, LSI significantly had a notably positive impact on reducing BMI for the participants with an initial BMI of less than 35.0 kg/m^2^ (DM = −1.82 kg/m^2^, 95% CI -2.91 to −0.72, n = 5, *p* = 0.001). This beneficial effect was also observed for more obese individuals with a BMI over 35.0 kg/m^2^ (DM = −1.56 kg/m^2^, 95% CI -2.93 to −0.19, n = 4, *p* = 0.025).

#### Waist circumference

The meta-analysis of nine studies with a total of 749 patients showed a beneficial effect (DM = −3.34 cm, 95% CI -5.06 to −1.63, df = 8; p = 0.001) on WC management. The evidence was graded as high quality.

#### Effect of LSI on blood-related parameters

The meta-analysis of five studies with a total of 183 patients showed no beneficial effect on blood glucose (DM = −0.19 mmoL/L, 95% CI -0.29 to −0.09, df = 4; *p* < 0.001), insulin (DM = −0.98 mU/L, 95% CI -2.23 to 0.28, df = 4; *p* = 0.127), HOMA-IR (DM = −0.01, 95% CI -0.45 to 0.43, df = 5; *p* = 0.974), testosterone (DM = −0.12 nmoL/L, 95% CI -0.02 to 0.23, df = 4; *p* = 0.024), and FAI index management (DM = −0.53, 95% CI -1.91 to 0.86, df = 2; *p* = 0.457), respectively. However, a beneficial effect was found for SHBG (DM = −5.55 nmoL/L, 95% CI -1.89 to −9.211, df = 4; *p* = 0.003). The quality of evidence for all parameters investigated was downgraded to moderate due to imprecision or moderate to high heterogeneity (Table 2).

#### Effect of LSI on ovulation, pregnancy, and live birth incidence

The meta-analysis of four studies with a total of 123 patients showed a beneficial effect of LSI on ovulation (OR = 11.23, 95% CI 2.51 to 50.23, df = 3; *p* = 0.002), pregnancy (OR = 1.49, 95% CI 1.04 to 2.15, df = 10; *p* = 0.032, I^2^ = 44%), and live births (OR = 1.51, 95% CI 0.92 to 2.47, df = 8; *p* = 0.099; I^2^ = 65%), respectively. The evidence for ovulation was downgraded from high to moderate due to the reported imprecision (sample size <300), while data on pregnancy were rated as high-quality evidence.

Table 3 shows the results of the meta-regression analysis for two categories of variables: (a) patient-related (initial age, weight, and BMI) and (b) training variables such as training volume (i.e., period, weekly frequency, total number of training sessions) and time spent in training (i.e., duration of a single training session). No significant predictors were found for weight reduction following LSI.

**Table 3:** Meta-regression for patient-related and training variables of different subscales to predict intervention effect on weight management.

	Coefficient	Standard error	95% lower CI	95% upper CI	*Z* value	*p* value
*Patient-related variables*						
Age (years)	−0.671	0.571	−1.4546	0.3857	−1.257	0.321
Weight (kg)	−0.0048	0.0137	−0.0316	0.0220	−0.35	0.7268
Body Mass Index (kg/m^2^)	−0.3257	0.3962	−1.1022	0.4509	−0.82	0.4111
*Training volume*						
Training period (weeks)	−0.0190	0.2256	−0.4611	0.4231	−0.08	0.9329
Training frequency (per week)	−0.6487	1.0057	−2.6197	1.3224	−0.65	0.6189
Total number of training sessions (per study)	−0.0194	0.0421	−0.1019	0.0631	−0.46	0.6443
*Time spent in training*						
Duration of a single training session (min)	0.0092	0.1423	−0.2698	0.2881	0.06	0.9486

Bolded values refer to the statistical significance of the observed results; the CI confidence interval.

#### Meta-regression analysis for patient-related and training variables in pregnancy

Table 4 shows the results of the meta-regression analysis for two categories of variables: (a) patient-related (initial age and BMI) and (b) training variables such as training volume (i.e., period, weekly frequency, and total number of training sessions) and time spent in training (i.e., duration of a single training session). It was found that a training period (weeks) is a predictor of successful pregnancy following LSI.

**Table 4:** Meta-regression for patient-related and training variables of different subscales to predict intervention effect on pregnancy.

	Coefficient	Standard error	95% lower CI	95% upper CI	*Z* value	*p* value
*Patient-related variables*						
Age (years)	−0.0341	0.1057	−0.2413	0.1731	0.47	0.6392
Weight (kg)	0.0251	0.0328	−0.0386	0.0952	0.57	0.563
Body Mass Index (kg/m2)	0.0381	0.0544	−0.0685	0.1447	0.70	0.4835
*Training volume*						
Training period (weeks)	−0.0718	0.0223	−0.1155	−0.0281	−3.22	**0.0013**
Training frequency (per week)	0.1259	0.1478	−0.1638	0.4157	0.85	0.3943
Total number of training sessions (per study)	−0.0029	0.0147	−0.0317	0.0259	−0.20	0.8432
*Time spent in training*						
Duration of a single training session (min)	0.0253	0.0289	−0.0314	0.0820	0.88	0.3815

Bolded values refer to the statistical significance of the observed results, the CI confidence interval.

## Discussion

In this systematic review and meta-analysis, 576 journals were screened, and 10 articles were selected. During an additional literature review conducted in December 2022, 498 journal articles were screened. Of these, five articles were selected for their qualitative insights on LSI and infertility intervention in overweight and obese women. Only a limited number of articles specifically explored the relationship between LSI, female infertility, and obesity. Furthermore, only a few studies followed the included subjects for a longer period of time so that long-term results on pregnancy and live births were also visible. The results of the present meta-analysis indicate that LSI can be an effective treatment for weight management, as evidenced by decreased weight (DM = −3.52 kg, *p* = 0.024), BMI (DM = −1.75 kg/m^2^, *p* < 0.001), and WC (DM = −3.34 cm, *p* = 0.001) after LSI. Moreover, we observed positive effects of LSI on increasing ovulation and pregnancy rates in overweight women with infertility. Furthermore, a meta-regression analysis showed no significant predictors among the related variables for the effect of LSI on weight management.

Our results are in line with previously published studies showing that LSI is efficient in reducing body weight (15, 33): for 12 of the 15 included studies, the reduction ranged from 0.7 to 12.9%, while in the control group, the average weight loss ranged from 0.1 to 8.4%. However, one study also found a weight gain (34). Additional subgroup analysis showed that the initial BMI of the participants was not a significant moderator of those effects (Q = 0.001; *p* = 0.980). On the contrary, the summarized effects of a pairwise comparison showed that the initial BMI of participants may influence the overall decrease in weight after LSI. Data processing was performed separately for two groups according to the initial BMI level, namely for subjects with a BMI below 35.0 kg / m^2^ and a BMI above 35.0 kg / m^2^. For subjects with a BMI below 35.0 kg / m^2^, the BMI decreased, but not significantly (DM = −3.62 kg, *p* = 0.109), while for the subjects in the group with a BMI above 35.0 kg / m^2^, the BMI decreased significantly (DM = −3.69 kg, *p* = 0.019). This raises the question of what kind of intervention and lifestyle change would be most appropriate for women with a BMI below 35.0 kg/m^2^. Moreover, the form of exercise (including type, volume, and intensity) and progression should probably be more frequent and/or intense for women with a BMI below 35.0 kg/ m^2^ than for women with a BMI above 35.0 kg/ m^2^, as tailored exercise could lead to a greater reduction in weight and BMI.

WC was reported in 9 of the 15 studies, and the meta-analytic approach showed a reduction of WC in the experimental group on average from 1.4 to 12.2%, while in the control group, there was an average reduction between 0.0 and 10.6%. Phy et al. previously showed that an 8-week and 12-week weight loss intervention in overweight and obese women with PCOS has an effect on reduction in WC and consequently improved insulin sensitivity, reduced testosterone, and improved fertility (35, 36).

Regular PA has been found to increase SHBG levels (37), and low serum SHBG levels are considered a relevant biomarker of abnormal metabolism and are related to insulin resistance and abnormalities in glucose and lipid metabolism (38). Previously published meta-analyses showed that LSI does impact SHBG, which was reported in five studies (20, 39–42).

In 4 of the 15 studies in the present meta-analysis, LSI was shown to have a beneficial effect on ovulation, and in 11 of the 15 studies on the pregnancy of overweight and obese women diagnosed with infertility. The LSI group had 11.23 times more ovulatory incidence than the control group, which in turn increased the ability to conceive. Furthermore, it was established that a 2–5% reduction in body weight has been associated with the restoration of ovulation (43). Accordingly, the proportion of pregnancies was higher in the intervention group, as almost a fifth of the subjects became pregnant, that is, 18.40%. In the LSI group, there was a higher proportion of live births: 17.83%.

Our findings are consistent with the systematic reviews already carried out. However, our analysis differs from the previous studies (7, 11, 15) because here we used the meta-analytic approach to identify various LSIs to explore their effects on fertility factors. LSI includes various components, and a variation was observed even in the length of LSI included in the presented MA. The mean length of LSI was 14.2 weeks, with the shortest intervention being 4 weeks (44) and the longest being 24 weeks (19, 45, 46), with 12 and 16 weeks being the most common durations. PA was part of the intervention in the fourteen studies included, but they were very differently defined. Ten LSI involved only aerobic exercises such as walking, brisk walking, jogging, or similar activity (18, 19, 39, 42, 44, 45, 47–50), two interventions involved structured exercise training (20, 41), one intervention included both aerobic and resistance training (51), and two did not specify the type of exercise intervention (34, 40). Three interventions with aerobic exercise were defined as 10,000 steps per day (19, 44, 48).

Pharmacological treatment was used in 2 intervention groups and 6 control groups out of the 15 included studies. In three of the 15 intervention groups and one control group, psychological or behavioral advice was provided to the subjects and performed by a health professional (34). Psychotherapy can be an important intervention that should be recommended for couples suffering from any form of infertility.

In addition to the intervention and control groups, 3 out of 15 studies had a second experimental group (20, 39, 47), and one intervention had an active control group (18). However, the present study’s focus was on comparing the LSI and control groups, even though several comparisons between studies were possible. At the same time, this might be one of the limitations of the present meta-analysis.

It should be mentioned that some of the selected studies had additional groups not addressed in the present analysis: 3 out of 15 studies had a third test group (20, 39, 47), and one intervention had a real control group (18). Since the focus of the present study was on comparing the LSI and control groups, it was decided that additional comparisons between studies were not performed. This is one of the limitations of the present meta-analysis.

When it comes to the issue of infertility, it is necessary to mention two aspects that were not known in the past, but their understanding and influence can help in comprehending the problem of infertility in the future. Scientists have determined that COVID-19 has had and continues to have an effect on the reproductive health of both women and men. Angiotensin-converting enzyme 2 (ACE2), a functional receptor for SARS-CoV-2, is a key component of the renin-angiotensin (SRA) system that modulates the cleavage of angiotensin II (Ang II) and Ang (1–7). Upon cell invasion, COVID-19 disrupts SRA by reducing ACE2 expression in host cells, leading to an increased Ang II inflammatory response (52). Ang II, ACE2, and Ang (1–7) regulate basic functions in the female and male reproductive systems. In women, this includes folliculogenesis, steroidogenesis, oocyte maturation, ovulation, and endometrial regeneration (53). According to the researchers, knowing the effect of the virus on fertility also changes and adapts infertility treatment (54), which slowed down a bit during the pandemic as clinics that perform artificial insemination procedures had stopped or limited treatment (55, 56).

Artificial intelligence (AI) has been widely applied in the field of reproductive health to enhance diagnosis, treatment, and overall healthcare delivery. Medenica et al. have found that AI has proven to be a very important and effective tool that will bring great innovation to the field of reproductive medicine. There are many ways in which artificial intelligence affects reproductive health: medical imaging and diagnostics (analyze medical images, MRIs, etc., to assist in detecting conditions), ART (AI can optimize and predict the success rate of IVF), customized and individualized treatment plans (based on patients’ medical histories and genetic information), and fertility tracking and predictions (to optimize timing for conception) (57, 58).

### Limitations and research recommendations

The advantage of the present research was the narrow and precise inclusion criteria, with which a small number of studies with comparable LSI were obtained. A meta-analysis was performed for each factor, using the PEDro scale to assess the reporting quality of randomized controlled trials and the TIDieR rating for reporting details of the intervention elements of a study.

The present review is limited because the studies and LSI parameters are very diverse. Consequently, the selection presented difficulties for comparison. Moreover, as there was a range of types of PA inside each LSI, it is not obvious which type of PA can improve fertility and better influence reproductive health. Only a few studies have defined PA as FITT (frequency, intensity, time or duration, and type). For future research, it is suggested that the PA with the acronym FITT be precisely defined, allowing other researchers to perform the exercise. In addition, it would be of great interest for research and practice to directly compare the effects of LSI on the body anthropometrics of subjects with BMIs above and below 35.0 kg/m^2^, as the results from the present meta-analysis showed inconclusive findings. Thus, these results must be interpreted with caution as the group comparison did not achieve a level of significance (Q = 0.001, *p* = 0.980), while pairwise comparisons did for women with BMI ≥35 kg/m^2^ (DM = −3.69 kg, *p* = 0.019) but not for those with less than 35 kg/m^2^ of the initial BMI (DM = −3.62 kg, *p* = 0.109). Thus, it is necessary to define the form and progression of the exercise, which probably should be different for women with a BMI above and below 35.0 kg/m^2^ when weight loss is a primary goal. Moreover, it would be of great interest to assess the adherence to an intervention in relation to LSI effectiveness in weight management. For future research, it is suggested that LSI be defined in greater detail, structured more carefully to suit the participants’ characteristics, and conducted over a longer period. The suggested modifications might lead to a greater effect of LSI, which consequently means that, in the case of appropriate findings, they could be implemented in practice and healthcare.

We would like to emphasize that an investigation of motor skills combined with BMI might provide further insights into the LS-fertility association and, in that sense, whether physical fitness parameters might be relevant biomarkers that can describe the risk of infertility.

## Conclusion

The findings of the present meta-analysis LSI (PA, diet, pharmacological treatment, or psychological advice) may have beneficial effects on some reproductive health outcomes in overweight and obese women with diagnosed infertility. The present meta-analysis showed that LSI has a beneficial effect on anthropometric measures (weight, BMI, and WC) and no beneficial effect on blood-related parameters, except SHBG. Moreover, the beneficial effects of the LSI were established as improved ovulation, a higher chance of pregnancy, and live births for overweight and obese infertile women.

## Data availability statement

The original contributions presented in the study are included in the article/supplementary material, further inquiries can be directed to the corresponding author.

## Author contributions

AS: Conceptualization, Data curation, Investigation, Project administration, Writing – original draft, Writing – review & editing. VH: Conceptualization, Writing – review & editing. CJWM: Data curation, Supervision, Validation, Visualization, Writing – review & editing. EA: Data curation, Validation, Writing – review & editing. MV: Funding acquisition, Resources, Validation, Writing – review & editing. TBP: Supervision, Validation, Writing – review & editing. AP: Conceptualization, Formal analysis, Investigation, Methodology, Software, Writing – original draft, Writing – review & editing, Data curation, Supervision, Validation.

## Abbreviations

BMI: body mass index
PCOS: polycystic ovary syndrome
LSI: lifestyle interventions
IVF: *in vitro* fertilization
WC: waist circumference
PA: physical activity
CI: confidence intervals
OR: odds ratio
